# Early Depression Independently of Other Neuropsychiatric Conditions, Influences Disability and Mortality after Stroke (Research Study—Part of PROPOLIS Study)

**DOI:** 10.3390/biomedicines8110509

**Published:** 2020-11-17

**Authors:** Katarzyna Kowalska, Łukasz Krzywoszański, Jakub Droś, Paulina Pasińska, Aleksander Wilk, Aleksandra Klimkowicz-Mrowiec

**Affiliations:** 1 Department of Neurology, Faculty of Medicine, Jagiellonian University Medical College, 31-008 Kraków, Poland; katarzyna.olga.kowalska@gmail.com; 2 Institute of Psychology, Pedagogical University of Krakow, 30-084 Kraków, Poland; lukasz.krzywoszanski@up.krakow.pl; 3 Doctoral School in Medical and Health Sciences, Jagiellonian University Medical College, 31-008 Kraków, Poland; jakub.dros@gmail.com; 4 Department of Medical Didactics, Faculty of Medicine, Jagiellonian University Medical College, 31-008 Kraków, Poland; paulina.potoczek@gmail.com; 5 Department of Neurosurgery, University Hospital, 30-688 Kraków, Poland; wialeksander@gmail.com

**Keywords:** post-stroke depression, disability level, mortality

## Abstract

Post-stroke depression (PSD) is the most frequent neuropsychiatric consequence of stroke. The nature of the relationship between PSD and mortality still remains unknown. One hypothesis is that PSD could be more frequent in those patients who are more vulnerable to physical disability, a mediator variable for higher level of physical damage related to higher risk of mortality. Therefore, the authors’ objective was to explore the assumption that PSD increases disability after stroke, and secondly, that mortality is higher among patients with PSD regardless of stroke severity and other neuropsychiatric conditions. We included 524 consecutive patients with acute stroke or transient ischemic attack, who were screened for depression between 7–10 days after stroke onset. Physical impairment and death were the outcomes measures at evaluation check points three and 12 months post-stroke. PSD independently increased the level of disability three (OR = 1.94, 95% CI 1.31–2.87, *p* = 0.001), and 12 months post-stroke (OR = 1.61, 95% CI 1.14–2.48, *p* = 0.009). PSD was also an independent risk factor for death three (OR = 5.68, 95% CI 1.58–20.37, *p* = 0.008) and 12 months after stroke (OR = 4.53, 95% CI 2.06–9.94, *p* = 0.001). Our study shows the negative impact of early PSD on the level of disability and survival rates during first year after stroke and supports the assumption that depression may act as an independent mediator for disability leading to death in patients who are more vulnerable for brain injury.

## 1. Introduction

Stroke is not only a leading cause of permanent functional disability, but also often causes severe impairment of mental health. Post-stroke depression (PSD) is the most frequent neuropsychiatric complication of stroke. In the meta-analysis by Hackett and Pickles [1], the pooled data showed that depression was present in 31% of stroke survivors at any time up to five-years post stroke, however its frequency varied across studies from 5% at two to five days after stroke to 84% at three months after stroke. Our data on PSD, among Polish patients with stroke, showed that PSD occurs in 54.58% of patients at the hospital, in 58.51% three months, and in 54.75% 12 months after the stroke [2].

It is important to recognize that depression is not a normal consequence of stroke, and still a lot of patients with stroke and physical impairment will not develop depression. Depression often coexist with other neuropsychiatric conditions which also increase the risk of negative prognosis, like apathy, anxiety, dementia, or delirium, and which often are misdiagnosed with depression. Sorting them out is essential for both, a correct risk assessment and for proper interventions.

Depressive symptoms occurring early after stroke increase the risk of negative consequences including death [3]. The rate of mortality among patients with PSD differs at different time points after stroke, also different risk factors are identified to increase the risk of death in this population [3,4,5]. Despite the fact that many studies have dealt with PSD, the nature of the relationship between PSD and mortality remains unknown and requires further analysis in order to draw a convincing conclusion. Among different hypothesis about the relationship between PSD and mortality one states that depression could be more frequent in those patients who are more vulnerable to physical disability [6] and PSD could act as a mediator variable for severe physical damage related to higher risk of mortality. A better understanding of this association would strengthen the evidence for causality, improve the therapeutic approach to patients with PSD, and provide prognostic information on survival. To check this hypothesis, we assumed that PSD negatively influences disability after stroke, regardless of stroke severity, other neuropsychiatric conditions, and higher mortality among patients with PSD.

Therefore, the objective of this study was to assess the change in the level of disability over a year in patients with PSD and their risk of death compared to depression-free patients by controlling other neuropsychiatric conditions and the severity of stroke.

## 2. Materials and Methods

This study was conducted as part of a larger prospective study, known as the PROPOLIS (PRospective Observational POLIsh Study on post-stroke delirium). Testing took place in the stroke unit at the University Hospital and Outpatient Clinic at the Neurology Department, University Hospital, Krakow. All procedures performed in this study involving human participants were in accordance with the ethical standards of the institutional and national research committee and with the 1964 Helsinki declaration and its later amendments. Informed written consent was provided by each participant or caregiver. The Local Bioethics Committee of Jagiellonian University approved the study (KBET/63/B/2014).

### 2.1. Population and Design

The consecutive patients admitted to the Stroke Unit at the University Hospital in Krakow, with stroke (ischemic or hemorrhagic) or transient ischemic attack (TIA) met inclusion criteria (Patients > 18 years of age, admitted within 48 h from the first stroke symptoms, speaking Polish, without serious communication deficits), were included into this sub-study. All patients had neuroimaging (CT/MRI) performed on admission. Stroke was defined as a sudden onset of neurological deficit lasting longer than 24 h. All patients were treated according to standard protocols of international guidelines [7].

Data regarding socio-demographic factors and comorbidities was collected. The Cumulative Illness Rating Scale (CIRS) was used as a general indicator of health status [8]. The severity of clinical deficit after stroke was graded by the National Institutes of Health Stroke Scale (NIHSS) [9] and the disability prior to admission was assessed by the modified Rankin Scale (mRS) [10].

Depression symptoms were assessed between 7 and 10 days after admission with Polish version of Patient Health Questionnaire-9 (PHQ-9) [11]. This questionnaire queries symptoms present using 4-point Likert scale with item scores ranging from 0 (symptoms not present) to 3 (symptoms present nearly every day). The score ranges from 0 (no depressive symptoms) to 27 (all symptoms occurring nearly every day) and can be used to determine depression severity (0–4 indicates no depression, 5–9 mild depression, 10 to 14 moderate depression, 15–19 moderately severe depression and 20–27 severe depression). PHQ-9 shows good reliability, validity and clinical utility when used in stroke patients [12]. Patients enrolled in the study completed the questionnaire on their own or with the help of a psychologist when filling out was impossible or difficult (e.g., the patient could not hold the pen because of a paresis or had a visual impairment). Depression was diagnosed if the patient received 5 or more points on the PHQ-9 scale [13].

To evaluate post-stroke apathy (PSA), the Apathy Evaluation Scale-C (AES-C) [14] was used. AES is an 18-item questionnaire with a clinician rated version that was applied in this study. The questions address patient’s activities, interest in doing things, relationship with others and feelings over the past two to three weeks. Each item is rated on a 4-point Likert scale with item scoring ranging from 1 (not at all true) to 4 (very true). The total AES-C score ranges from 18 to 72, with higher scores indicating greater apathy. The AES has good reliability and validity and was frequently used in studies on post-stroke apathy [14]. Apathy was diagnosed with AES score of ≥ 37 points [15].

Anxiety was measured with Polish adaptation of State Trait Anxiety Inventory (STAI) [16,17], the 40-item instrument, measuring, respectively, transient and enduring levels of anxiety. The state scale used in the present study administered as a self-completion questionnaire by the interviewer, assessed how the patients felt at the moment or in the recent past and how they anticipate their feelings to be in a specific, hypothetic situation in the future. The STAI scale is scored on four levels of anxiety intensity from 1 (not at all) to 4 (very much) and with a sum score between 20 and 80. The raw results are interpreted by referring to a relevant sten scores and then categorized into three levels of anxiety: low (1–4 sten), moderate (5–6 sten) and high (7–10 sten) [17].

Patients were screened for delirium with the abbreviated version of Confusion Assessment Method (bCAM) or the Intensive Care Units version (CAM-ICU), specifically in patients with motor aphasia or those who could not communicate for other reasons [18,19]. The final diagnosis of delirium was based on both clinical observation and structural assessment. The diagnostic criteria for delirium were based on the DSM-5 classification [20].

To screen for pre-stroke depression (pre-SD), a member of family/spouse or a close informant of the patient’s household filled out the Neuropsychiatric Inventory [21]. In addition, patients were asked about previous treatment for depression, and medical records were checked for antidepressants among the medications currently taken by the patient.

In order to diagnose patients with pre-stroke dementia, a Polish version of Informant Questionnaire on Cognitive Decline in the Elderly (IQCODE) was used [22].

### 2.2. Outcome Assessment

We assessed the following outcome measures: presence of depression between 7–10th day after admission to the hospital, degree of disability in daily activities and mortality after stroke 3 and 12 months after stroke during the follow-up visit. Patients who did not attend a follow-up visit were contacted by phone and the information was gathered. A neurologist and a psychologist, both uninvolved in the baseline assessment of patients, were responsible for data acquisition.

### 2.3. Statistics

Statistical analysis was performed using Statistica 13.3 software (StatSoft^®^, Kraków, Poland). Qualitative variables were compared using the chi-squared test with or without Yates’ correction, as appropriate. Quantitative values were presented as medians with interquartile ranges (IQRs) and compared with the Mann-Whitney U test due to non-normal distribution in each case. Correlations were statistically evaluated using Pearson’s correlation tests and correlation coefficients (r) were obtained.

Associations between PSD (based on the PHQ-9 cut-off point) and 3-month and 12-month mortality were found using univariate logistic regression models. Predictive values were presented as odds ratios (ORs) with 95% confidence intervals (CIs). Similarly, associations between PSD and disability (increase in mRS of ≥1) were evaluated. Then, multivariate logistic regression models were adjusted for age, gender, and comorbidities (CIRS score). *p*-values < 0.05 were considered statistically significant.

## 3. Results

**Figure 1:**
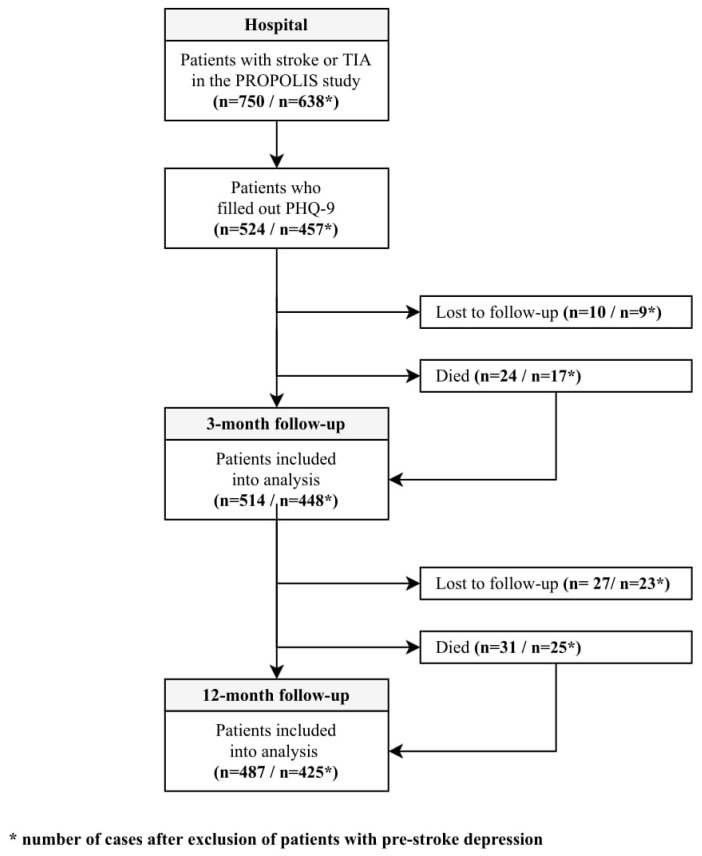
Study flowchart.

**Figure 2:**
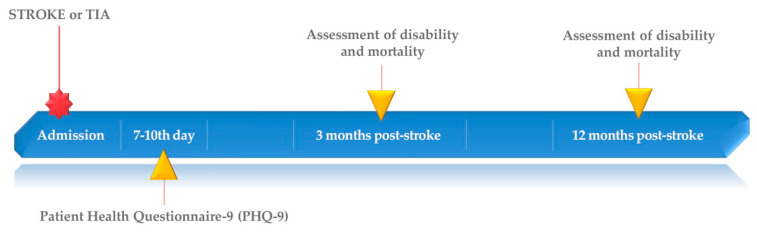
Study timeline.

From 750 patients included into PROPOLIS study 524 filled out the PHQ-9. After three and 12 months after stroke 514 and 487 patients were available for examination, respectively. A flowchart (Figure 1) and a timeline (Figure 2) show the study design.

When compared to controls, patients with PSD were significantly older, more often females, less often had left hemispheres stroke and treatment with recombinant tissue plasminogen activator (rt-PA), suffered from pneumonia, and had higher C-reactive protein (CRP) levels during hospitalization. Also, they were significantly more physically disabled prior to admission, more often had TIA or stroke in the past and had more comorbidities at baseline comparisons. Early depression was significantly more often accompanied by other neuropsychiatric conditions: apathy, anxiety, delirium, and dementia. Table 1 shows the details.

After three months, 24 patients died, 10 were lost from the follow-up and mRS score was not obtained in 18 patients. After 12 months, 31 persons died, and another 27 patients were lost from the follow-up. Patients who were lost from the follow-up did not differ significantly from those analyzed. Table 2 shows the results.

**Figure 3:**
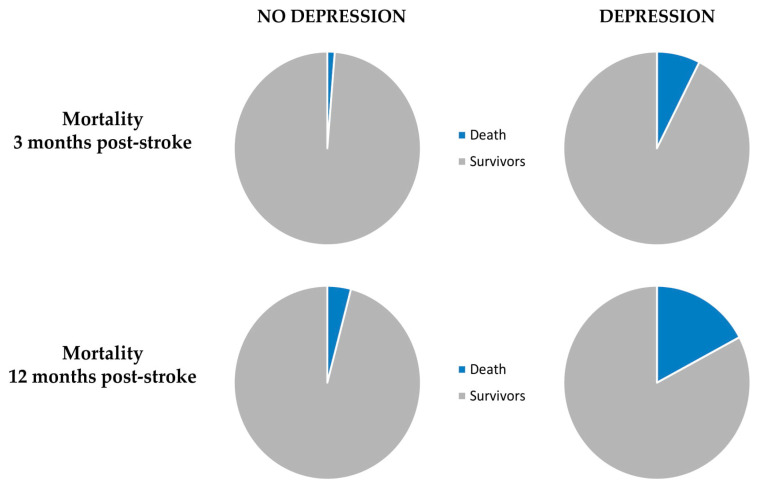
A pie chart presenting the influence of post-stroke depression on mortality.

**Figure 4:**
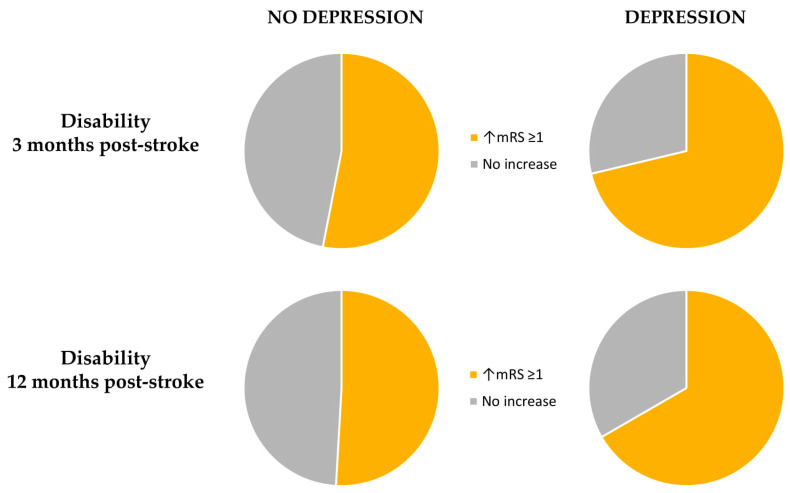
A pie chart presenting the influence of post-stroke depression on disability.

In the first step, we compared patients with PHQ-9 ≥ 5 points with those who scored 4 or less. After 3 and 12 months after stroke, PSD was an independent risk factor for death in multivariable logistic regression analysis. Also, PSD independently increased the level of disability of 1 point on mRS among patients with PSD three and 12 months post-stroke. Table 3 and Figure 3 and Figure 4 show the final results.

In the second step, we excluded patients with pre-SD from further analyses. The general characteristic of patients with PSD and controls, after exclusion of patients with pre-SD, are shown in Table 4. The final results were very similar to those obtained in first analysis. Only the side of stroke lost its significance.

In regression analyses, PSD was still an independent variable for mortality and increased level of disability measured by mRS three and 12 months after stroke. Table 5 shows the results.

Patients that were lost from the follow-up in this sub-analysis did not differ significantly from analyzed group. Table 6 shows the details.

In the third step, we compared only pre-SD with patients without depression (pre- or post-stroke). Patients with pre-SD were significantly more often women, had more comorbidities and had higher level of disability prior to admission. Table 7 shows the details.

Pre-SD increased the level of disability on mRS of 1 point at threeand 12 months post-stroke and predicted mortality within 12 months after stroke. Table 8 shows the results.

Patients with pre-SD significantly more often had PSD and they also had significantly more severe depression when compared to other individuals. There was no relationship between NIHSS score and PHQ-9 score. Table 9 and Table 10 show the results.

## 4. Discussion

In our cohort, depression was diagnosed in 54.58% of patients between seven and 10 days after stroke. Patients who developed depressive symptoms in acute phase of stroke had about six times higher risk of death three months after stroke and nearly 4.5 times higher risk after 12 months, when compared to patients without depression. PSD negatively influenced level of disability and mortality rate at three and 12 months after stroke. Both outcomes were independent from stroke severity and concomitant neuropsychiatric conditions.

Other studies have also reported an association between PSD and mortality after stroke. In study by Williams et al. [23], among total of 51,119 patients hospitalized with an ischemic stroke, those diagnosed with PSD had a higher three-year mortality risk, even despite being younger and having fewer chronic conditions. Previous meta-analysis [4,5,6], also showed that mortality was an independent outcome of depression after stroke and patients with early PSD had a risk of death about 1.5 higher as compared with non-depressed individuals, considering both short- and long-term mortality. In a study by Razmara et al. [24], the combination of depression and stroke was associated with all—cause mortality, with the highest risk of death in those aged 65–74 years. Patients with depressive symptoms were about 35 times more likely to die when compared to stroke survivors without depression.

Our study found that PSD increases the level of disability both three and 12 months after stroke. In earlier studies [25,26], depressed patients have been found more dependent in activities of daily living at three and 15-month follow-up than patients without depression. Paolucci et al. [27] estimated that PSD is a relevant factor that is responsible for about 15% of the increased disability observed in post—stroke depressive patient.

As was shown, pre-SD was associated with higher stroke morbidity and mortality [28]. In our cohort, pre-SD was independently related to increased mortality 12 months post-stroke but not three months. The number of patients with pre-SD was small which can explain this lack of association for the three-month observation.

Pre-SD, which is due to many factors, e.g., social, degenerative, or vascular, also negatively influenced the level of disability both three and 12 months after stroke. Results of this study suggest, that regardless of etiology, depression increases negative outcomes after stroke.

The association between stroke and depression is well established as well as between stroke and poor functional outcome. The connecting factor between depression, physical impairment, and mortality in patients with stroke can be brain-derived neurotrophic factor (BDNF), a member of the neurotrophin family, involved in neuronal development, differentiation, and survival.

There is a general agreement that etiology of mood disorders is multifactorial. Hypotheses about the participation and interrelationship of down regulation of neurotrophins, inflammation, hypothalamic-pituitary-adrenal axis hyperactivity and stress in pathophysiology of depression have an important support in literature [29].

Recent findings have reported that BDNF is a key regulator in the neuro-immune axis regulation, but its potential mechanism in depression remains unclear [30]. Lower BDNF levels were found to be a significant risk factor for PSD [31] as well as in clinically depressed individuals [32]. BDNF could intermediate between depression and the level of disability after stroke. Stroke activates microglia, which are brain guards and the first non-neuronal cells to respond to various acute brain injuries [33]. An inflammatory state can contribute to the development and progression of depression pathology, influencing alterations of the neuroplasticity caused by reduced BDNF expression, activity, and affinity to a receptor [30,34,35]. Moreover, BDNF levels are mediated by physical exercise enhancing its levels in the brain [36]. Activity-driven increases in BDNF have also been shown to promote motor recovery after stroke [37]. Physical rehabilitation may be impaired by depressions, and depressed patients are less likely to exercise what lowers the level of BDNF and intensify functional impairment. For the time being, there is not enough evidence of a definitive link between BDNF and depression, disability and mortality, and their potential interrelationships need to be confirmed in future studies.

Immunological mechanisms, as mentioned, are implicated in the pathogenesis of depressive symptoms. C-reactive protein is the inflammatory biomarker, an acute phase protein that increases in level during the acute phase of inflammation. Patients with depression exhibit increased peripheral blood concentrations of CRP [38,39]. Elevated CRP along with other peripheral blood markers of inflammation have been found to predict development of depression [40] and resistance to antidepressant therapy [41]. A few studies have examined the relationship between circulating CRP and risk of post-stroke depression with conflicting results [42,43,44]. In the previous sub-study, we found that this association was significant for depression diagnosed during hospitalization, but there was no association between depression diagnosed three months post-stroke and CRP levels [45]. Interestingly, in this present, much larger study, patients with depression, diagnosed at the hospital, had significantly higher level of CRP than dementia-free patients, thus supporting the hypothesis of the role of immunological mechanisms in development of depressive symptoms.

In the pathophysiology of depression, a dysregulated kynurenine pathway has also been implicated. In this pathway, tryptophan is broken down into kynurenine and then to neurotoxic quinolinic acid and decreases the availability of tryptophan for serotonin synthesis. The altered levels of kynurenines have been implicated in psychiatric [46] and neurodegenerative diseases [47]. Preliminary data from one small study among patients with stroke also suggest that the kynurenine pathway may be implicated in PSD and disability [48]. Kynurenic acid seems to be useful not only in process of diagnosis but also in prediction of the treatment response [49].

Research shows that inflammation is an important, multi-directional factor in the etiology of depression, but further research is still needed on its role in diagnosing depression, guiding decision making on clinical treatment and monitoring the course of the disease and the risk of its relapse.

### Strengths and Weaknesses of the Study

The first step in arriving at a correct diagnosis of mental health problems is to distinguish depression from other psychiatric syndromes that can cause confusion, such as delirium, dementia, apathy, or anxiety. Evaluating different mental problems concurrently is also important to distinguish between the right diagnoses, given the overlap between them. Careful and broad evaluation of mental health problems at the hospital is a strong side of PROPOLIS.

Prior psychiatric illness can influence mental status post-stroke, i.e., represents either recurrence or continuation of a preexisting psychiatric illness. Therefore, in PROPOLIS, we carefully screened for neuro-psychiatric conditions including depression, dementia, delirium, anxiety, and apathy pre-stroke.

This study had prospective design and included a large number of patients at the baseline, which helped to sustain a reasonably large number of patients during all follow-ups. Patients that were lost in the follow-up didn’t differ significantly from those followed-up.

A variety of raters; neurologist and psychologist assessed patients at baseline and during follow-up visits. This is considered as the strength of this study, because follow-up raters were blind for the patients’ previous performance and behavior. On the other hand, patients who are more familiar to assessors are more willing to ask for help if they have problems with understanding the questions from the questionnaire and therefore provide more adequate answers. Therefore, a variety of raters can be also considered as a weakness of the study.

Some limitations of our study and bias inducers should also be addressed. Firstly, the PROPOLIS was designed to determine frequency, predictors, and clinical consequences of post-stroke delirium. Depressive symptoms were considered as a secondary endpoint of the study. Secondly, we used questionnaires to describe symptoms of depression, since using interviews with mental health professional was not feasible. Thirdly, the first evaluation for depressive disorders took place before the 14th day after stroke, which may have overestimated the prevalence of depression in the acute phase of stroke. Fourthly, during the follow-up visits, we observed, most depressed patients did not have formal diagnosis of depression and were not treated, but data on the treatment with antidepressants were not collected during the follow-ups. Because treatment with antidepressants might influence the study outcome, this is considered as a limitation. Fifth, as this was a single center study, the generalizability of our results may be limited.

## 5. Conclusions

Depression can act as a mediator variable for a higher disability level and mortality in patients more vulnerable to brain injury, independently of other neuropsychiatric mental health problems.

A high prevalence of depression after stroke should stress the need for future research exploring its possible pathomechanism and testing, if an early management of depression may change life expectancy after stroke and improve the outcome, even if functional deficits remain.

## Figures and Tables

**Table 1:** Baseline characteristics of patients without and with post-stroke depression in hospital. (All included patients).

Variable	Data	No Depression *n* = 238 (45.42%)	Depression *n* = 286 (54.58%)	*p*-Value
Male gender *	524	143/238 (60.08)	122/286 (42.66)	<0.001
Age [years] **	524	68 (60–78)	71 (62–80)	0.022
Higher education *	518	49/235 (20.85)	49/283 (17.31)	0.306
Length of education [years] **	515	11 (10–14)	11 (10–13)	0.122
Hemorrhagic stroke *	524	12/238 (5.04)	16/286 (5.59)	0.780
TOAST classification:				
- large-artery atherosclerosis *	457	27/210 (12.86)	28/247 (11.34)	0.618
- cardioembolism *	457	11/210 (5.24)	15/247 (6.07)	0.701
- small-vessel occlusion *	457	63/210 (30.00)	79/247 (31.98)	0.648
- other determined etiology *	457	107/210 (50.95)	122/247 (49.39)	0.740
- undetermined etiology *	457	2/210 (0.95)	3/247 (1.21)	0.855
Side of stroke:				
- right hemisphere *	524	93/238 (39.09)	136/286 (47.55)	0.051
- left hemisphere *	524	112/238 (47.06)	105/286 (36.71)	0.017
- posterior part *	524	31/238 (13.03)	35/286 (12.24)	0.787
- more than one localization *	524	2/238 (0.84)	10/286 (3.50)	0.084
rt-Pa treatment *	524	68/238 (28.57)	52/286 (18.18)	0.007
Thrombectomy *	524	12/238 (5.04)	12/286 (4.20)	0.645
Medical history:				
- hypertension *	524	158/238 (66.39)	206/286 (72.03)	0.163
- diabetes *	524	49/238 (20.59)	92/286 (32.17)	0.003
- atrial fibrillation *	524	39/238 (16.39)	54/286 (18.88)	0.457
- myocardial infraction *	524	33/238 (13.87)	40/286 (13.99)	0.968
- PCI or CABG *	524	22/238 (9.24)	25/286 (8.74)	0.841
- smoking—ever *	523	118/237 (49.79)	149/286 (52.10)	0.599
- smoking—current *	523	62/237 (26.16)	86/286 (30.07)	0.323
- previous stroke or TIA *	522	32/237 (13.50)	60/285 (21.05)	0.024
CIRS, total score **	524	7 (5–11)	10 (6–13)	<0.001
Pneumonia *	524	8/238 (3.36)	23/286 (8.04)	0.038
Urinary tract infections *	505	58/232 (25.00)	81/273 (29.67)	0.242
Length of hospital stay [days] **	524	9 (8–10)	9 (8–11)	0.320
Aphasia in hospital *	524	53/238 (22.27)	50/286 (17.48)	0.170
Neglect in hospital *	524	22/238 (9.24)	34/286 (11.89)	0.329
Vision deficits in hospital *	524	59/238 (24.79)	89/286 (31.12)	0.109
Delirium in hospital*	524	25/238 (10.50)	66/286 (23.08)	<0.001
AES score at 7–10th day in hospital **	480	29 (20–38)	34 (25–43)	<0.001
STAI-S score at 7–10th day in hospital **	520	32 (27–39)	42 (33–52)	<0.001
STAI-T score at 7–10th day in hospital **	519	35 (30–41.5)	47 (40–54)	<0.001
NIHSS at admission **	524	4 (2–8)	4 (2–9)	0.649
Pre-hospital mRS **	524	0 (0–0)	0 (0–1)	0.002
Pre-hospital IQCODE **	436	78 (78–79)	78 (78–83)	0.007
CRP level in hospital [mg/l] **	507	3.82 (1.63–10.34)	5.75 (2.04–18.60)	0.003

* *n* (%); ** median (IQR); TOAST—Trial of Org 10172 in Acute Stroke Treatment; rt-Pa—recombinant tissue plasminogen activator; PCI—percutaneous coronary interventions; CABG—coronary artery bypass graft; TIA—transient ischemic attack; CIRS—Cumulative Illness Rating Scale; AES—Apathy Evaluation Scale; STAI—State-Trait Anxiety Inventory (S—state scale, T—trait scale); NIHSS—National Institutes of Health Stroke Scale; mRS—Modified Rankin Scale; IQCODE—Informant Questionnaire on Cognitive Decline in the Elderly; CRP—C-reactive protein.

**Table 2:** Comparison of analyzed and lost to follow-up cases. (All included patients).

**Variable**	**Data**	**Analyzed Cases**	**Lost to Follow-Up**	***p*-Value**
**Comparison of Analyzed (*n* = 514) and Lost to Follow-Up (*n* = 10) Cases for 3-Month Mortality**				
Male gender *	524	259/514 (50.39)	6/10 (60.00)	0.777
Age (years) **	524	69 (61–79)	69.5 (62–77)	0.581
CIRS, total score **	524	8.5 (5–12)	7 (5–10)	0.441
NIHSS at admission **	524	4 (2–9)	3 (0–10)	0.428
Pre-hospital mRS **	524	0 (0–0)	0 (0–0)	0.834
**Comparison of Analyzed (*n* = 496) and Lost to Follow-Up (*n* = 28) Cases for 3-Month mRS**				
Male gender *	524	250/496 (50.40)	15/28 (53.57)	0.744
Age (years) **	524	69 (61–79)	71 (62–79.5)	0.662
CIRS, total score **	524	9 (5–12)	7.5 (5–11.5)	0.374
NIHSS at admission **	524	4 (2–9)	3 (0–7)	0.113
Pre-hospital mRS **	524	0 (0–0)	0 (0–0.5)	0.858
**Comparison of Analyzed (*n* = 487) and Lost to Follow-Up (*n* = 37) Cases for 12-Month Mortality and mRS**				
Male gender *	524	244/487 (50.10)	21/37 (56.76)	0.435
Age (years) **	524	69 (61–79)	71 (63–78)	0.639
CIRS, total score **	524	9 (5–12)	7 (4–11)	0.130
NIHSS at admission **	524	4 (2–8)	4 (2–12)	0.317
Pre-hospital mRS **	524	0 (0–0)	0 (0–0)	0.871

* *n* (%); ** median (IQR); CIRS—Cumulative Illness Rating Scale; NIHSS—National Institutes of Health Stroke Scale; mRS—Modified Rankin Scale.

**Table 3:** Influence of post-stroke depression on mortality and disability 3 and 12 months after stroke. (All included patients).

		**Incidence, *n* (%)**		**Univariate Logistic Regression Model**		Multivariate Logistic Regression Model *	
**Variable**	**Data**	**No Depression**	**Depression**	**OR (95CI)**	***p*-Value**	**OR (95CI)**	***p*-Value**
**3 months:**							
Mortality	514	3/234 (1.28)	21/280 (7.50)	6.243 (1.838–21.204)	0.003	5.685 (1.586–20.378)	0.008
Increase in mRS of ≥1	496	121/228 (53.07)	191/268 (71.27)	2.194 (1.514–3.179)	<0.001	1.944 (1.315–2.876)	0.001
**12 months:**							
Mortality	487	9/220 (4.09)	46/267 (17.23)	4.880 (2.331–10.216)	<0.001	4.535 (2.065–9.964)	<0.001
Increase in mRS of ≥1	487	112/220 (50.91)	178/267 (66.67)	1.929 (1.336–2.783)	<0.001	1.681 (1.141–2.478)	0.009

* adjusted for age, gender and CIRS (Cumulative Illness Rating Scale); mRS—Modified Rankin Scale.

**Table 4:** Baseline characteristics of patients without and with post-stroke depression in hospital. (Only patients without Pre-Stroke Depression).

Variable	Data	No Depression *n* = 223 (48.80%)	Depression *n* = 234 (51.20%)	*p*-Value
Male gender *	457	139/223 (62.33)	104/234 (44.44)	<0.001
Age (years) **	457	67 (60–78)	73 (62–80)	0.011
Higher education *	451	47/220 (21.36)	40/231 (17.32)	0.276
Length of education (years) **	449	12 (10–14)	11 (9–13)	0.064
Hemorrhagic stroke *	457	12/223 (5.38)	12/234 (5.13)	0.904
TOAST classification:				
- large-artery atherosclerosis *	398	24/197 (12.18)	22/201 (10.95)	0.699
- cardioembolism *	398	11/197 (5.58)	11/201 (5.47)	0.961
- small-vessel occlusion *	398	59/197 (29.95)	65/201 (32.34)	0.607
- other determined etiology *	398	101 (51.27)	101/201 (50.25)	0.839
- undetermined etiology *	398	2/197 (1.02)	2/201 (1.00)	0.630
Side of stroke:				
- right hemisphere *	457	88/223 (39.46)	108/234 (46.15)	0.149
- left hemisphere *	457	102/223 (45.74)	88/234 (37.61)	0.079
- posterior part *	457	31/223 (13.90)	30/234 (12.82)	0.734
- more than one localization *	457	2/223 (0.90)	8/23 (3.42)	0.128
rt-Pa treatment *	457	61/223 (27.35)	41/234 (17.52)	0.012
Thrombectomy *	457	11/223 (4.93)	9/234 (3.85)	0.570
Medical history:				
- hypertension *	457	148/223 (66.37)	172/234 (73.50)	0.096
- diabetes *	457	44/223 (19.73)	76/234 (32.48)	0.002
- atrial fibrillation *	457	35/223 (15.70)	44/234 (18.80)	0.380
- myocardial infraction *	457	32/223 (14.35)	32/234 (13.68)	0.836
- PCI or CABG *	457	22/223 (9.87)	20/234 (8.55)	0.626
- smoking—ever *	456	112/222 (50.45)	118/234 (50.43)	0.996
- smoking—current *	456	59/222 (26.58)	66/234 (28.21)	0.697
- previous stroke or TIA *	455	30/222 (13.51)	49/233 (21.03)	0.036
CIRS, total score **	457	7 (4–11)	9 (6–12)	<0.001
Pneumonia *	457	7/223 (3.14)	21/234 (8.97)	0.009
Urinary tract infections *	442	52/218 (23.85)	68/224 (30.36)	0.124
Length of hospital stay [days] **	457	9 (8–10)	9 (8–11)	0.472
Aphasia in hospital *	457	48/223 (21.52)	40/234 (17.09)	0.230
Neglect in hospital *	457	20/223 (8.97)	28/234 (11.97)	0.296
Vision deficits in hospital *	457	53/223 (23.77)	71/234 (30.34)	0.114
Delirium in hospital *	457	20/223 (8.97)	53/234 (22.65)	<0.001
AES score at 7–10th day in hospital **	411	28 (20–36)	32.5 (24–42)	<0.001
STAI-S score at 7–10th day in hospital **	453	31 (27–39)	41 (32–51)	<0.001
STAI-T score at 7–10th day in hospital **	452	35 (30–41)	45 (39–53)	<0.001
NIHSS at admission **	457	4 (2–7)	4 (2–9)	0.673
Pre-hospital mRS **	457	0 (0–0)	0 (0–1)	0.003
Pre-hospital IQCODE **	377	78 (78–79)	78 (78–81)	0.019
CRP level in hospital [mg/L] **	442	3.74 (1.59–10.77)	5.44 (1.97–17.25)	0.010

* *n* (%); ** median (IQR); TOAST—Trial of Org 10172 in Acute Stroke Treatment; rt-Pa—recombinant tissue plasminogen activator; PCI—percutaneous coronary interventions; CABG—coronary artery bypass graft; TIA—transient ischemic attack; CIRS—Cumulative Illness Rating Scale; AES—Apathy Evaluation Scale; STAI—State-Trait Anxiety Inventory (S—state scale, T—trait scale); NIHSS—National Institutes of Health Stroke Scale; mRS—Modified Rankin Scale; IQCODE—Informant Questionnaire on Cognitive Decline in the Elderly; CRP—C-reactive protein.

**Table 5:** Influence of post-stroke depression on mortality and disability 3 and 12 months after stroke. (Only patients without Pre-stroke depression).

		**Incidence, *n* (%)**		**Univariate Logistic Regression Model**		Multivariate Logistic Regression Model *	
**Variable**	**Data**	**No Depression**	**Depression**	**OR (95CI)**	***p*-Value**	**OR (95CI)**	***p*-Value**
**3 months:**							
Mortality	448	3/219 (1.37)	14/229 (6.11)	4.688 (1.328–16.548)	0.016	4.447 (1.184–16.707)	0.027
Increase in mRS of ≥1	432	109/213 (51.17)	151/219 (68.95)	2.119 (1.431–3.137)	<0.001	1.856 (1.227–2.806)	0.003
**12 months:**							
Mortality	425	9/208 (4.33)	33/217 (15.21)	3.966 (1.847–8.512)	<0.001	3.712 (1.644–8.381)	0.002
Increase in mRS of ≥1	425	104/208 (50.00)	140/217 (64.52)	1.818 (1.232–2.682)	0.003	1.588 (1.056–2.387)	0.026

* adjusted for age, gender and CIRS (Cumulative Illness Rating Scale); mRS—Modified Rankin Scale.

**Table 6:** Comparison of analyzed and lost to follow-up cases. (Only patients without Pre-Stroke Depression).

**Variable**	**Data**	**Analyzed Cases**	**Lost to Follow-Up**	***p*-Value**
**Comparison of Analyzed (*n* = 448) and Lost to Follow-Up (*n* = 9) Cases for 3-Month Mortality**				
Male gender *	457	237/448 (52.90)	5/9 (66.67)	0.629
Age (years) **	457	69.5 (61–79)	68 (62–71)	0.366
CIRS, total score **	457	8 (5–12)	6 (5–10)	0.276
NIHSS at admission **	457	4 (2–8)	3 (0–10)	0.496
Pre-hospital mRS **	457	0 (0–0)	0 (0–0)	0.991
**Comparison of Analyzed (*n* = 432) and Lost to Follow-Up (*n* = 25) Cases for 3-Month mRS**				
Male gender *	457	229/432 (53.01)	14/25 (56.00)	0.771
Age (years) **	457	69 (61–79)	71 (62–78)	0.909
CIRS, total score **	457	8 (5–12)	6 (5–10)	0.278
NIHSS at admission **	457	4 (2–8)	3 (0–7)	0.216
Pre-hospital mRS **	457	0 (0–0)	0 (0–1)	0.573
**Comparison of Analyzed (*n* = 425) and Lost to Follow-Up (*n* = 32) Cases for 12-Month Mortality and mRS**				
Male gender *	457	223/425 (52.47)	20/32 (62.50)	0.273
Age (years) **	457	69 (61–79)	69.5 (62–78)	0.874
CIRS, total score **	457	8 (5–12)	7 (3–11)	0.122
NIHSS at admission **	457	4 (2–8)	4 (2–12)	0.364
Pre-hospital mRS **	457	0 (0–0)	0 (0–0)	0.575

* *n* (%); ** median (IQR); CIRS—Cumulative Illness Rating Scale; NIHSS—National Institutes of Health Stroke Scale; mRS—Modified Rankin Scale.

**Table 7:** Baseline characteristics of patients without depression and with pre-stroke depression.

Variable	Data	No Depression *n* = 223 (79.08%)	Pre-Stroke Depression *n* = 59 (20.92%)	*p*-Value
Male gender *	282	139/223 (62.33)	20/59 (33.90)	<0.001
Age (years) **	282	67 (60–78)	68 (61–79)	0.302
Previous stroke or TIA *	281	30/222 (13.51)	13/59 (22.03)	0.106
CIRS, total score **	282	7 (4–11)	11 (6–15)	<0.001
NIHSS at admission **	282	4 (2–7)	5 (2–11)	0.264
Pre-hospital mRS **	282	0 (0–0)	0 (0–1)	0.034

* *n* (%); ** median (IQR); TIA—transient ischemic attack; CIRS—Cumulative Illness Rating Scale; NIHSS—National. Institutes of Health Stroke Scale; mRS—Modified Rankin Scale.

**Table 8:** Influence of pre-stroke depression on mortality and disability 3 and 12 months after stroke.

		**Incidence, *n* (%)**		**Univariate Logistic Regression Model**		Multivariate Logistic Regression Model *	
**Variable**	**Data**	**No Depression**	**Pre-Stroke Depression**	**OR (95CI)**	***p*-Value**	**OR (95CI)**	***p*-Value**
**3 months:**							
Mortality	277	3/219 (1.37)	6/58 (10.34)	8.308 (2.011–34.322)	0.003	2.414 (0.376–15.497)	0.353
Increase in mRS of ≥1	270	109/213 (51.17)	47/57 (82.46)	4.484 (2.153–9.338)	<0.001	3.965 (1.826–8.610)	<0.001
**12 months:**							
Mortality	264	9/208 (4.33)	12/56 (21.43)	6.030 (2.394–15.191)	<0.001	3.406 (1.064–10.904)	0.039
Increase in mRS of ≥1	264	104/208 (50.00)	42/56 (75.00)	3.000 (1.546–5.823)	0.001	2.395 (1.171–4.897)	0.017

* adjusted for age, gender and CIRS (Cumulative Illness Rating Scale); mRS—Modified Rankin Scale.

**Table 9:** Association of incidence of pre-stroke depression with post-stroke depression and median PHQ-9 score.

Variable	Data	Pre-Stroke Depression *n* = 59 (20.92%)	No Pre-Stroke Depression *n* = 457 (88.57%)	*p*-Value
Post-stroke depression *	516	48/59 (81.36)	234/457 (51.20)	<0.001
PHQ-9 score **	516	9 (6–12)	5 (2–9)	<0.001

* *n* (%); ** median (IQR); PHQ-9—The Patient Health Questionnaire-9.

**Table 10:** Correlations between NIHSS and PHQ-9 at the hospital.

Group	Data	Pearson’s Correlation Coefficient (r)	*p*-Value
All patients	524	−0.0128	0.770
Pre-stroke depression excluded	457	−0.0384	0.413

NIHSS—National Institutes of Health Stroke Scale; PHQ-9—The Patient Health Questionnaire-9.
